# Panton-Valentine Leucocidin (PVL) as a Potential Indicator for Prevalence, Duration, and Severity of *Staphylococcus aureus* Osteomyelitis

**DOI:** 10.3389/fmicb.2017.02355

**Published:** 2017-11-28

**Authors:** Bei Jiang, Yinan Wang, Zihan Feng, Lei Xu, Li Tan, Shuang Zhao, Yali Gong, Cheng Zhang, Xiaoqiang Luo, Shu Li, Xiancai Rao, Yizhi Peng, Zhao Xie, Xiaomei Hu

**Affiliations:** ^1^State Key Laboratory of Trauma, Burns and Combined Injury, Institute of Burn Research, Southwest Hospital, Third Military Medical University, Chongqing, China; ^2^Department of Microbiology, College of Basic Medical Sciences, Third Military Medical University, Chongqing, China; ^3^Cadet Brigade, Third Military Medical University, Chongqing, China; ^4^Department of Orthopedics, Southwest Hospital, Third Military Medical University, Chongqing, China

**Keywords:** *Staphylococcus aureus*, Panton-Valentine leucocidin (PVL), osteomyelitis, molecular epidemiology, virulence factor

## Abstract

*Staphylococcus aureus* is the most common cause of the difficult-to-treat osteomyelitis (OM). To better diagnose and manage *S. aureus* OM, especially for severe and long duration cases, indicators for risk prediction and severity evaluation are needed. Here, 139 clinical *S. aureus* isolates from orthopedic infections were divided into OM group (60 isolates from 60 OM patients) and non-OM group (79 isolates from 79 non-OM patients). Molecular types, antimicrobial susceptibility, and virulence factor profiles were evaluated and compared between the two groups to identify potential indicators associated with the prevalence of *S. aureus* OM. Clinical manifestations and laboratory data were analyzed to identify indicators affecting OM duration and severity. We found that some sequence types were specific to OM infection. The *pvl*, *bbp*, and *ebps* genes were associated with *S. aureus* OM prevalence. The *pvl*, *bbp*, and *sei* genes were associated with relatively longer OM duration. Panton-Valentine leucocidin (PVL)-positive *S. aureus* OM presented more serious inflammatory responses. Our results emphasize the significance of PVL in affecting the prevalence, duration, and severity of *S. aureus* OM. Diagnosing and monitoring PVL-related *S. aureus* OM may help direct better prognosis and treatment of these patients.

## Introduction

Osteomyelitis (OM) is a serious infectious disease in orthopedics, with an inflammatory and destructive process on bone and its surrounding tissues including marrow, cortex, and periosteum (Lew and Waldvogel, 2004). The high recurrence rate, long duration time, and serious bone destruction of OM bring huge physical and economic burdens to these patients (Lew and Waldvogel, 2004; Kalinka et al., 2014). *Staphylococcus aureus*, which is notorious for its strong adaptive capacity to adverse environments and the ability of elaborating diverse virulence factors (Otto, 2014), is the most common pathogenic bacteria of OM (Lew and Waldvogel, 2004). The diagnosis of *S. aureus* OM relies on a combination of clinical symptoms, microbiology, histopathology, laboratory data, and imaging examinations (Lew and Waldvogel, 2004). Although biopsy of bone tissue or marrow is not always appropriate or feasible for OM patients, the microbiological cultures of sinus-tract or wound specimens from the OM infection sites of these patients are also useful for pathogenic diagnosis, especially when a *S. aureus* strain is isolated (Mackowiak et al., 1978). To solve the problem of the difficult-to-treat *S. aureus* OM, most attention have been paid on therapeutic approaches including inadequate debridement and antimicrobial usage, few focused on bacteriological factors involved.

Several previous studies attempted to seek for virulence factors associated with prevalence of *S. aureus* OM. The *bbp* gene (encoding bone sialoprotein binding protein) was considered to be associated with OM (Tung et al., 2000), but different studies presented opposite conclusions (Tristan et al., 2003; Kalinka et al., 2014). One study suggested that the *bbp* gene was associated more with OM/arthritis than with endocarditis (Tristan et al., 2003), while another found that the *bbp* gene played no role in OM (Kalinka et al., 2014). The *fnbB* gene was also once reported to be more frequently carried in patients with *S. aureus* OM, but the sample size was small in that study (only 21 OM isolates, 10 sepsis isolates and nasal colonization isolates) (Kalinka et al., 2014). Panton-Valentine leucocidin (PVL) is a pore-forming toxin composed of two components, LukS-PV and LukF-PV, which constitute a heptamer, leading to lysis of neutrophil, monocytes, and macrophages (Bakthavatchalam et al., 2017; Takadama et al., 2017). PVL received great attention because it is thought to be an important toxin of *S. aureus* and an epidemiological biomarker of severe *S. aureus* infections in several studies (Vandenesch et al., 2003; Kaneko and Kamio, 2004; Sheikh et al., 2015). For example, PVL-positive *S. aureus* strains are more likely to cause severe necrotizing pneumonias and severe skin and soft tissue infections (Morgan, 2007; Bakthavatchalam et al., 2017). They were reported more frequently isolated from young patients (Muttaiyah et al., 2010). However, the significance of PVL is controversial because other studies challenged this opinion (Shallcross et al., 2013). For example, several clinical trials show that severe SSTIs caused by PVL-positive and PVL-negative strains do not result in different outcomes (Tong et al., 2015). A meta-analysis involved in 76 studies from 31 countries found that *pvl* genes were comparatively rare in invasive disease with poor prognosis (Shallcross et al., 2013). For OM infections, in a rabbit OM model, PVL was proposed to play a role in the persistence and rapid local extension of OM caused by community-acquired methicillin-resistant *S. aureus* (CA-MRSA) (Cremieux et al., 2009). In the United States, PVL-positive *S. aureus* accounted for up to two-thirds of *S. aureus*-related acute pediatric hematogenous OM (Bocchini et al., 2006; Sdougkos et al., 2007). As PVL-positive United States-300 clone is widely spread in the United States, it is difficult to separate the effects of United States-300 and those truly caused by the *pvl* gene (Sheikh et al., 2015). This can be avoided in China because of low prevalence of United States-300 (Song et al., 2013).

One of the most formidable challenges of OM therapy is its refractoriness and recurrence during long duration time which can continue for several decades (Lew and Waldvogel, 2004; Rao et al., 2011; Wang et al., 2017), but few studies focused on the microbiological factors affecting OM duration. Kalinka et al. (Kalinka et al., 2014) attempted to find differences on virulence factor profiles between *S. aureus* OM isolates from 11 patients with less than 2-month duration and 10 patients with more than 12-month duration, but no significant difference was found.

In consideration of controversial viewpoints on microbiological risk factors associated with *S. aureus* OM and few data on the relationship of molecular epidemiology and clinical manifestations of *S. aureus* OM, we conducted this study to explore microbiological factors that may affect the prevalence, duration, and severity of *S. aureus* OM. The results will provide information for risk factors of *S. aureus* OM, identify potential biomarkers for early diagnosis and warning for long duration and serious *S. aureus* OM.

## Materials and Methods

### Ethics Statement

The study was approved by the Committee of the First Affiliated Hospital of Third Military Medical University, China. No written informed consent was required because we received anonymized isolate samples. All the personal information was removed and was not present in the data of this study.

### Definitions

Patients were confirmed as *S. aureus* OM when all the following diagnostic items were fulfilled, including clinical symptoms, microbiology, histopathology, laboratory studies, and imaging examinations (Lew and Waldvogel, 2004). Isolates from the sites of *S. aureus* OM infection were assigned to OM group. Isolates from the contemporaneous inpatients at the center who had never been diagnosed as OM until enrollment were assigned to non-OM group. OM duration was defined as the duration time between the first and the last diagnosis of OM when the isolates were obtained for this study. A *S. aureus* clone comprising >10% of all isolates in a group was considered predominant clone (Cheng et al., 2013). An isolate was considered multidrug resistant (MDR) strain when it was resistant to three or more classes of non-β-lactam antimicrobials (Pillar et al., 2008). Interpretive standards for antimicrobials susceptibility were in accordance with the Clinical and Laboratory Standards Institute (CLSI)-2017 guidelines.

### Patients and Study Design

This cross-sectional study was conducted at the orthopedic center of Southwest Hospital, a tertiary hospital in southwest China. From September 2013 to September 2015, all the available *S. aureus* isolates (a total of 162 isolates) from inpatients at this center were enrolled into this study. All isolates were divided into OM and non-OM groups according to their corresponding clinical diagnosis (Lew and Waldvogel, 2004). For strains from the same patient with the same molecular types and virulence factors profile, only the earliest isolate was involved to maximally avoid a second enrollment of the same strain. The remaining 139 *S. aureus* isolates, including 60 isolates from 60 OM patients (OM group) and 79 isolates from 79 non-OM patients (non-OM group) were finally enrolled in this study. The undermentioned microbiological data were compared between two groups to seek for microbiological risk factors of *S. aureus* OM. Patients’ clinical manifestations and laboratory data, combining with the corresponding microbiological data were analyzed to find out factors affecting *S. aureus* OM duration and severity.

### Microbiological Data

Isolates of OM group (*n* = 60) were obtained from tissues (bone tissues or inflammatory granulation tissues from OM infection sites, *n* = 21), wounds (wounds infection close to the OM infection sites, *n* = 21), bone marrow (*n* = 11), and pus (sampled from sinus-tract of OM infection sites, *n* = 7). Isolates of non-OM group (*n* = 79) were obtained from infected wounds (*n* = 31), tissues (*n* = 23), pus (*n* = 15), blood (*n* = 5), and catheters (*n* = 5) of the contemporaneous inpatients. All the isolates were phenotypically identified to genus level by phenotypic methods (API staphy system, Biomerieux). MRSA was confirmed by *femB* and *mecA* duplex polymerase chain reaction (PCR) (Kobayashi et al., 1994). Staphylococcal chromosomal cassette *mec* (*SCCmec*) type of MRSA, multilocus sequence type (MLST), *spa* type, and *agr* group were determined as previously described (Shopsin et al., 1999; Enright et al., 2000; Gilot et al., 2002).

The presence of 36 virulence factors, including 11 adhension-associated genes (*bbp*, *ebps*, *cna*, *eno*, *icaA*, *icaD*, *fnbA*, *fnbB*, *fib*, *clfA*, and *clfB*) and 25 exotoxin-associated genes (*pvl*, *tst*, *eta*, *etb*, *edin*, *psm*α,*lukM*, *lukED*, *hla*, *hlb*, *hld*, *hlg*, *hlgv*, *sea*, *seb*, *sec*, *sed*, *see*, *seg*, *seh*, *sei*, *sej*, *sem*, *sen*, and *seo*), were detected by multiplex PCR in seven groups. Primers used for PCR of virulence factors were listed in Supplementary Table S1.

MicroScan system (Dade Behring, West Sacramento, CA, United States) was used to determine antimicrobial susceptibilities of 15 antimicrobials, including oxacillin (OXA), penicillin (PEN), nitrofurantoin (NIT), trimethoprim/sulfamethoxazole (SXT), erythromycin (ERY), ciprofloxacin (CIP), clindamycin (CLI), rifampicin (RIF), linezolid (LNZ), moxifloxacin (MFX), gentamicin (GEN), tetracycline (TCY), tigecycline (TGC), vancomycin (VAN), and levofloxacin (LVX). Drug resistance was determined according to CLSI guidelines.

### Clinical and Laboratory Data

All patients’ demographic information and basic diseases were collected. Peak values of C-reactive protein (CRP), erythrocyte sedimentation rate (ESR), white blood cell count (WBC), and absolute neutrophil count (ANC) before an operation intervention were collected from all the patients enrolled. Of note, CRP values of patients with basic inflammatory diseases or recent trauma history were excluded for their extremely and non-specifically high values (Neumaier et al., 2015). To identify indicators affecting OM duration, all the OM patients’ duration time was collected, and was divided into subgroups of more than or less than 24 months, and more than or less than 20 years. In the latter grouping method, only the OM patients older than 20 years old were included.

### Statistical Analysis

All statistical analyses were performed using SPSS 19.0 (Chicago, IL, United States) and GraphPad Prism 6.0 software (San Diego, CA, United States). Categorical and continuous variables were compared using χ^2^ test (with Yates’ continuity correction if necessary) and Student’s *t*-test, respectively. Demographic information and factors exhibited significant differences between OM and non-OM groups were subsequently assessed by univariate logistical regression analyses. Demographic information (sex and age) and variables with *p* < 0.2 in the univariate analyses were included in the covariates of multivariate logistic regression. *p* < 0.05 was considered to be statistically significant.

## Results

### Diverse Sources of Isolates Exist in Both Groups and Some Sequence Types Are Specific to *S. aureus* OM

**FIGURE 1:**
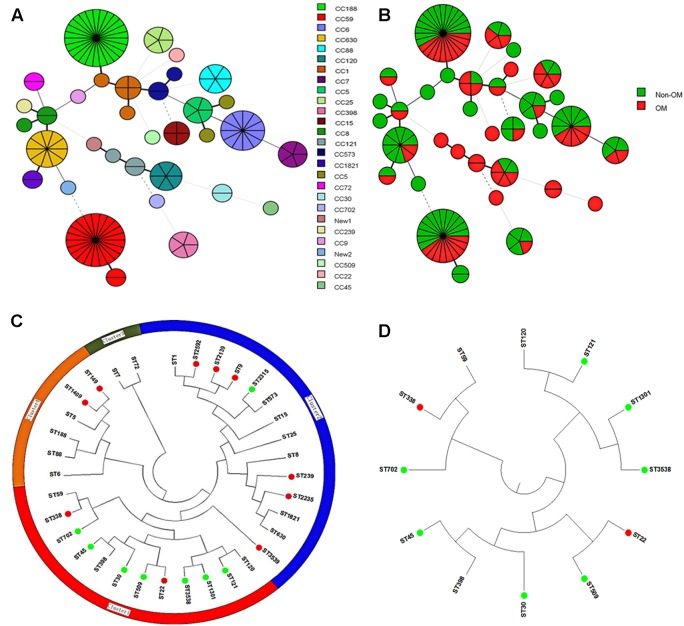
BioNumerics visualization and phylogenetic analysis of sequence types (STs). **(A,B)** STs stratified by clonal complexes (CCs) and OM/non-OM group were visually presented using BioNumerics. **(C)** Phylogenetic analysis of all the identified STs. All the STs were mainly divided into four clusters. (Green dot indicates STs specific to OM group; red dot indicates STs specific to non-OM group). Almost all the STs specific to OM were grouped to cluster 1. **(D)** Subtree of cluster 1 is shown separately.

To determine whether specific molecular type is responsible for *S. aureus* OM infection, we carried out multiple molecular typing (**Table 1**). MRSA detection showed that totally 28 MRSA isolates were identified, no difference in the proportion of MRSA isolates was found between OM and non-OM groups, and almost all of them belonged to *SCCmec* I or IV. MLST and *spa* types exhibited high diversity in both groups. Twenty-three sequence types (STs) belonged to 20 clonal complexes (CCs) and a singleton were identified in OM group. Twenty-five STs belonged to 19 CCs and a singleton were identified in non-OM group. One new ST was, respectively, identified in OM group (ST3538) and non-OM group (ST3539). ST188 and ST59 were predominant in both groups (11/60, 18.3% and 15/79, 19.0% for ST188; 9/60, 15.0% and 13/79, 16.5% for ST59). Meanwhile, ST188 and ST59 also, respectively, presented the most common STs of methicillin susceptible *Staphylococcus aureus* (MSSA) and MRSA, which was consistent with a previous investigation in China (Qiao et al., 2014). All the STs found in this study were stratified by CCs and OM/non-OM group, and visually presented using BioNumerics version 7.6 (**Figures 1A,B**). Phylogenetic analysis revealed that some STs were specific to OM infection and almost all of them grouped to cluster 1 (**Figures 1C,D**). Twenty-three *spa* types including three new types (t16342, t16519, and t16520) and 34 *spa* types including one new type (t16521) were identified in OM and non-OM groups, respectively. In both groups, t189 and t437 were the predominant *spa* types (10/60, 16.7% and 14/79, 17.7% for t189; 8/60, 13.3% and 12/79, 15.2% for t437). *agr* group I was determined to be the most common *agr* group in both groups (38/60, 63.3% and 62/79, 78.5%), and all the predominant STs and *spa* types mentioned above belonged to *agr* group I. *agr* group III and IV *S. aureus* were positively associated with OM group (*p* = 0.0040) (**Table 1**). All the new STs and *spa* types found in this study had been submitted to the database^1^^,^^2^ and assigned as new type names mentioned above.

**Table 1:** Comparison of molecular typing between osteomyelitis (OM) and non-OM groups.

	OM (*n* = 60)	Non-OM (*n* = 79)	*p*-value
MRSA, *n* (%)	9 (15.0)	19 (24.1)	0.19
*SCCmec* type of MRSA			
Type I, *n* (%)	2 (3.3)	12 (15.2)	0.11
Type IV, *n* (%)	7 (11.7)	6 (7.6)	0.060
Type V, *n* (%)	0 (0)	1 (1.3)	0.68
MLST			
Predominant type (*n*, %)	ST188 (11,18.3), ST59 (9, 15.0)	ST188 (15,19.0), ST59 (13, 16.5)	0.92, 0.82
New type (*n*)	ST3538 (1)	ST3539 (1)	
*spa* typing			
Predominant type (*n*, %)	t189 (10,16.7), t437 (8, 13.3)	t189 (14, 17.7), t437 (12, 15.2)	0.87, 0.76
New type (*n*)	t16342 (1), t16519 (1), and t16520 (1)	t16521 (1)	
*agr* grouping (*n*, %)			
Predominant group	*agr* I (38, 63.3)	*agr* I (62, 78.5)	**0.049**
*agr* III/IV	18 (30.0)	8 (10.1)	**0.0029**
Predominant clone (*n*, %)	ST188-t189-*agr* I (10, 16.7)	ST188-t189-*agr* I (13, 16.5)	0.97
	ST59-t437-*agr* I (8,13.3)	ST59-t437-*agr* I (11, 13.9)	0.92


*Significant differences are in boldface.*

### Antimicrobial Resistance Is Not Responsible for the Prevalence or Duration of *S. aureus* OM

**FIGURE 2:**
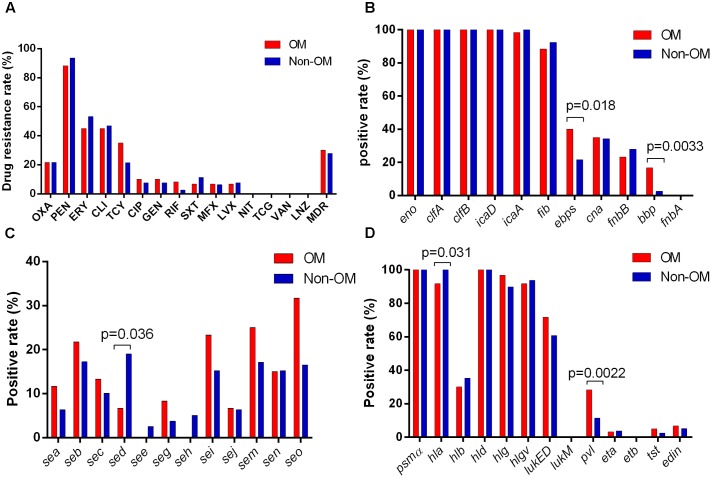
Antimicrobial susceptibility and virulence profiles of *Staphylococcus aureus* isolates. **(A)** Results of antimicrobial susceptibility tests. Positive rates of adhension-associated toxins **(B)**, enterotoxins **(C)**, and other exotoxins **(D)** are separately shown.

No significant antimicrobials resistance difference was found neither between OM and non-OM groups nor between OM patients with more than and less than 24 months/20 years durations. Eighteen and 22 MDR strains were detected in OM and non-OM groups, respectively, with no significant difference. Several antimicrobials presented relatively higher resistance rates in both groups including penicillin (88.3 and 93.7%), erythromycin (45 and 53.2%), clindamycin (45 and 46.9%), tetracycline (35 and 21.5%), and oxacillin (21.7 and 26.6%). While no resistant strain was found for linezolid, tigecycline, and vancomycin (**Figure 2A**).

### The *pvl*, *bbp*, and *ebps* Genes Are Positively Associated with Prevalence of *S. aureus* OM

To identify if any virulence factor plays as a potential indicator of *S. aureus* OM, 36 virulence factors of *S. aureus* were tested by PCR. The results showed that all isolates exhibited carriage of at least six adhension-associated and three exotoxin-associated virulence genes. The *eno*, *clfA*, *clfB*, *icaD*, *hld*, and *psm*α genes were detected in all isolates. Other virulence genes detected among more than 50% in both OM and non-OM groups included *icaA* (98.3 and 100%), *fib* (88.3 and 92.4%), *hla* (91.7 and 100%), *hlg* (96.7 and 89.9%), *hlgv* (91.7 and 93.7%), and *lukED* (71.7 and 60.8%). The *fnbA*, *etb*, and *lukM* genes were not detected in any isolate. We found a significant positive association of the *pvl* (*p* = 0.0022), *bbp* (*p* = 0.0033), and *ebps* (coding elastin binding protein) (*p* = 0.018) genes with OM isolates, whereas the *hla* (*p* = 0.031) and *sed* (*p* = 0.036) genes were negatively associated with OM isolates (**Figures 2B–D**). Univariate analysis revealed that the *pvl* (*p* = 0.014), *bbp* (*p* = 0.010), and *ebps* (*p* = 0.019) genes were significantly associated with the prevalence of OM, while the *sed* gene (*p* = 0.045) presented association with non-OM group. Remarkably, multivariate analysis revealed that the *pvl* gene independently played as a risk factor of *S. aureus* OM [17/60 (28.3%) versus 9/79 (11.4%); adjusted OR, 3.12; 95% CI, 1.04–9.35; *p* = 0.042] (**Table 2**).

**Table 2:** Univariate and multivariate logistic regression analyses of factors affecting OM prevalence.

	OM (*n* = 60)	Non-OM (*n* = 79)	Univariate analysis OR (95% CI), *P*	Multivariate analysis OR (95% CI), *P*
Age, median (range)	41 (5–72)	44 (5–75)	0.984 (0.963–1.006), 0.154	0.987 (0.964–1.012), 0.312
Male, *n* (%)	47 (78.3)	53 (67.1)	0.564 (0.560–1.221), 0.146	0.509 (0.216–1.197), 0.122
*agr* III/IV, *n* (%)	18 (30.0)	8 (10.1)	3.804 (1.522–9.507), **0.004**	1.276 (0.377–4.326), 0.695
*pvl*, *n* (%)	17 (28.3)	9 (11.4)	3.075 (1.259–7.509), **0.014**	3.021 (1.016–8.984), **0.047**
*bbp*, *n* (%)	10 (16.7)	2 (2.5)	7.700(1.619–36.619), **0.010**	2.540 (0.325–19.842), 0.374
*ebps*, *n* (%)	24 (40.0)	17 (21.5)	2.431 (1.155–5.120), **0.019**	1.737 (0.702–4.295), 0.232
*sed*, *n* (%)	4 (6.7)	15 (19.0)	0.305 (0.096–0.972), **0.045**	0.298 (0.088–1.009), 0.052
*hla*, *n* (%)	55 (91.7)	79 (100.0)	0(0), 0.999	–


*Demographic information and factors presented significant differences between two groups were assessed by logistical regression analyses. Demographic information and variables with *p* < 0.2 in the univariate analyses were included in the multivariate logistic regression. OR, odds ratio; CI, confidence interval. Significant differences are in boldface.*

### The *pvl*, *bbp*, and *sei* Genes Are Positively Associated with Duration of *S. aureus* OM, PVL-Positive *S. aureus* OM Present More Severe Inflammatory Responses

**FIGURE 3:**
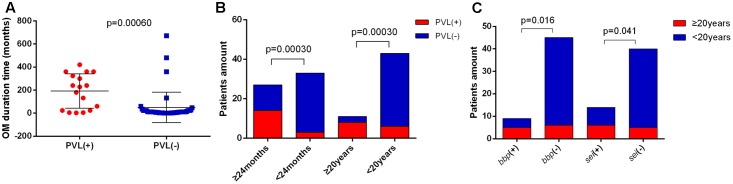
Indicators associated with *S. aureus* OM duration. **(A)** Patients with Panton-Valentine leucocidin (PVL)-positive *S. aureus* OM exhibited significantly longer OM duration time (average of 192.1 months vs. 50.0 months). The bars are expressed as the mean ± SEM. **(B)** The *pvl* gene was positively associated with OM duration of more than 24 months and 20 years. **(C)** The *bbp* and *sei* genes were positively associated with OM duration of more than 20 years.

Long duration and serious bone destruction are the greatest concerns in OM, so we attempted to find out factors affecting duration and severity of OM by analyzing the clinical manifestations of OM cases combining with their corresponding microbiological characteristics. We found that the OM duration time was significantly longer in PVL-positive patients (average of 192.1 months versus 50.0 months, *p* = 0.00060) (**Figure 3A**). Moreover, the *pvl* gene was positively associated both with OM duration of more than 24 months (*p* = 0.00030) and 20 years (*p* = 0.00030) (**Figure 3B**). The *bbp* (*p* = 0.016) and *sei* (*p* = 0.041) genes also contributed to OM duration of longer than 20 years (**Figure 3C**). In addition, all the patients with OM duration longer than 24 months carried MSSA, further suggesting that virulence factors rather than drug resistance were more important for longer OM duration. No other significance of specific clone types or antimicrobial resistance patterns was found between patients with relatively longer and shorter OM durations.

**FIGURE 4:**
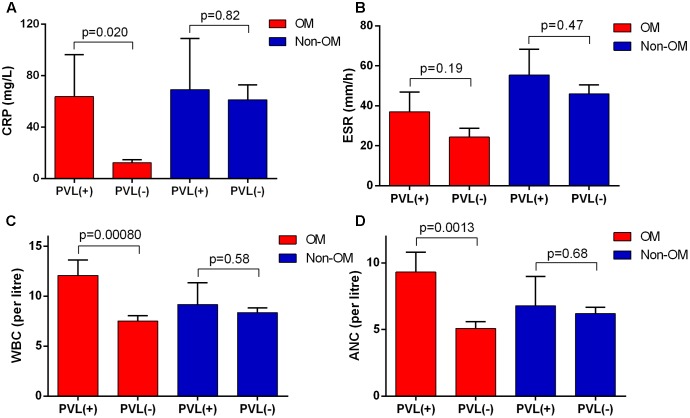
Panton-Valentine leucocidin-positive patients in OM group showed more severe inflammatory responses. The severity was reflected by **(A)** C-reactive protein (CRP), **(B)** erythrocyte sedimentation rate (ESR), **(C)** white blood cell count (WBC), and **(D)** absolute neutrophil count (ANC). The bars are expressed as the mean ± SEM.

In PVL-positive *S. aureus* OM patients, the values of CRP, ESR, WBC, and ANC all presented higher average values compared to the PVL-negative ones. Except for ESR, other three data showed significantly higher values in PVL-positive patients, indicating more severe inflammatory responses. While in non-OM group, no significant difference of these values was found between PVL-positive and PVL-negative patients (**Figures 4A–D**). No significant association was found between other virulence factors and these clinical laboratory data.

### PVL-Positive OM Cases and Isolates Presented Unique Clinical and Epidemiological Characteristics

**FIGURE 5:**
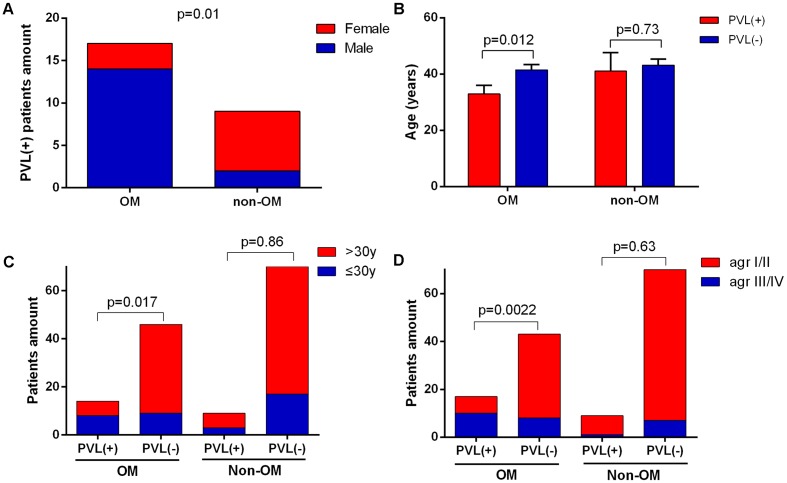
Characteristics of PVL-positive cases and isolates. PVL-positive cases in the OM group were more likely to be male **(A)** and younger **(B)** compared to the non-OM group. The bars are expressed as the mean ± SEM. Patients amount of PVL-positive OM was significantly higher amongst patients of less than 30 years old **(C)**. *agr* group III/IV isolates were more likely to carry *pvl* gene in OM group **(D)**.

In the view of great importance of *pvl* gene in *S. aureus* OM suggested above, we next investigated the features of PVL-positive OM cases and their corresponding isolates. The PVL-positive isolates were more tended to infect male patients in OM group compared to non-OM group, with proportions of 14/17 (82.4%) versus 2/9 (22.2%) (*p* = 0.01) (**Figure 5A**). Patients infected with PVL-positive *S. aureus* in OM group were younger than PVL-negative ones, with average ages of 32.3 versus 41.5 (*p* = 0.012). While the data were 43.2 versus 41 (*p* = 0.73) in non-OM group (**Figure 5B**). Similarly, the amount of patients who were younger than 30 years old were significantly more in PVL-positive OM compared to PVL-negative OM (*p* = 0.017), while this significant difference was not found amongst non-OM patients (*p* = 0.86) (**Figure 5C**). MLST types of PVL-positive strains in OM group were diverse and distributed on various positions of the phylogenetic tree (**Figure 1C**), including ST1, ST120, ST121, ST25, ST30, ST3538, ST398, ST59, ST6, and ST88, which indicated various sources of *pvl* gene. *agr* group III/IV strains accounted for 10/17 (58.8%) PVL-positive isolates in OM group, which was significantly more compared to the PVL-negative ones (8/43, 18.6%) (*p* = 0.0022). However, this significant difference was not found amongst PVL-positive isolates in non-OM group (1/9, 11.1% versus 7/70, 10.0%) (**Figure 5D**).

## Discussion

Although pathogenic microorganism, especially *S. aureus*, is the primary cause of OM (Lew and Waldvogel, 2004), the relationship between microbiological molecular epidemiology and clinical characteristics of OM is largely unknown.

It is not well known if any specific molecular types of *S. aureus* are associated with OM infection. Some studies indicated higher prevalence of MRSA in OM patients (Gadepalli et al., 2009). In this study, multiple molecular typing revealed that neither MRSA/MSSA, nor specific *spa* types were more likely to cause *S. aureus* OM. We found that STs in some cluster seem to be specific for OM infection (**Figures 1C,D**), and *agr* group III/IV may play as a risk factor of *S. aureus* OM (*p* = 0.0040). MLST is principally employed genetic typing method. It well reflects the underlying genealogical information and provides an accurate assessment of species and even strains, and therefore widely used in evolutionary and population studies for pathogens (Maiden, 2006). The clustering of some STs that are related to OM infections suggests that some genetic factors of theses STs make them more susceptible to OM infections. More detailed mechanisms underlying this phenomenon need further in-depth investigation, and would be meaningful for understanding the relationship between bacteria genetic makeup and infectious susceptibility. The significant difference amongst *agr* groups may be attributed to some virulence factors usually carried by specific *agr* group as previously reported (Xiong et al., 2009). Actually, in this study, the *bbp*-carrying isolates all belonged to *agr* group III/IV, and *agr* group III/IV isolates were more likely to carry *pvl* gene (*p* = 0.00020).

Antimicrobial resistance is becoming a serious public health problem, especially for ESKAPE pathogens (*Enterococcus faecium*, *S. aureus*, *Klebsiella pneumoniae*, *Acinetobacter baumannii*, *Pseudomonas aeruginosa*, and *Enterobacteriaceae*) (Rice, 2010; Cinquin et al., 2015). Antimicrobial resistance may cause recurrent infection and prolonged treatment duration (Lew and Waldvogel, 2004; Proctor et al., 2006). However, no specific antimicrobial resistance profile was found to be associated with prevalence or longer duration of *S. aureus* OM in this study. Several antimicrobials showed relatively high resistance rate in both groups. Amongst them, clindamycin, one of the preferred choices for treatment of *S. aureus* OM (Feigin et al., 1975; Bamberger and Boyd, 2005), presented a resistance rate of 45% in OM group. This may form a potential challenge to anti-infection therapy of *S. aureus* OM.

Previous studies had suggested that several virulence factors might contribute to *S. aureus* OM, but controversies exist. In the current study, we found that the *pvl* gene was associated with *S. aureus* OM in both univariate analysis (*p* = 0.014) and multivariate analysis (*p* = 0.042). This result is partly consistent with the investigation in *S. aureus*-related pediatric acute hematogenous OM in the United States (Bocchini et al., 2006; Dohin et al., 2007), and further indicates the significant role of PVL in the prevalence of *S. aureus* OM. PVL was few related to bone-associated infections previously, although the contribution of PVL to clinical diseases remains controversial by now (Bakthavatchalam et al., 2017), the relationship between PVL and bone-associated infections deserves more attention and needs more basic experimental studies. The *S. aureus* Bbp protein is predicted to be important in the localization of bacteria to bone tissues, and thus related to the pathogenicity of OM (Tung et al., 2000). In this study, we also found that the *bbp* gene was associated with *S. aureus* OM, but only in the univariate analysis (*p* = 0.010), not in the multivariate analysis (*p* = 0.374). Our results support the view of Tristan et al.’s (2003) study, but contrary to Kalinka et al.’s (2014) results. More studies combining clinical investigation and basic research are needed to clearly illustrate the role of Bbp in *S. aureus* OM. The *ebps* gene was first implied as a risk factor of *S. aureus* OM (*p* = 0.019) in this study. However, in consideration that the *ebps* gene could often be detected in the *bbp*-positive isolates, especially in some specific STs, such as ST121 and ST30 (Aung et al., 2011; Montanaro et al., 2016), and all the *bbp*-positive isolates in this study carried the *ebps* gene. So, the significantly higher carrying rate of the *ebps* gene in OM group may be related to the carriage of the *bbp* gene. Although *fnbB* gene was once indicated to be associated with *S. aureus* OM (Kalinka et al., 2014), no significantly higher prevalence of *fnbB* gene or other virulence factors were observed in OM isolates. The *sed* gene was found significantly more frequent in non-OM group (*p* = 0.045) in this study. This was partly because more ST59 clone, which was reported tending to carry *sed* gene (Yu et al., 2015), was involved in non-OM group.

One of the most concerned issues in OM is the long duration time, which makes the cure difficult and brings heavy burn to both patients and the society (Lew and Waldvogel, 2004). It is widely reported that clinical treatment in the course of OM has an important influence on OM duration (Lew and Waldvogel, 2004; Rao et al., 2011). In other infections, some microbiological factors had been related to infection durations (Lindberg et al., 2000; Nowrouzian et al., 2011), while microbiological factors were seldom investigated in OM. A study with only 21 *S. aureus* OM patients of different durations found no significant difference in virulence factors (Kalinka et al., 2014). The current study suggested that the prevalence of *pvl* gene was significantly higher in patients with OM duration of more than 24 months (*p* = 0.0003) and 20 years (*p* = 0.00030) (**Figure 3B**). Furthermore, we first proposed that the carriage of *bbp* (*p* = 0.016) or *sei* gene (*p* = 0.041) might also increase the risk of long duration OM with more than 20 years duration (**Figure 3C**). The *bbp* gene had not been related to infection duration previously. Because, Bbp may be related to bacteria colonization (Tung et al., 2000), so it is reasonable to result in longer infection time in OM infections, but to make this conclusion more convincing, more epidemiological studies should be conducted in the future. Several previous studies suggested that *egc* cluster (including *seg*, *sei*, *sem*, *sen*, and *seo*) might be responsible for long-term and persistent infections (Lindberg et al., 2000; Nowrouzian et al., 2011), but we did not find significant differences on other members of *egc* cluster between OM patients with different durations in this study. These results suggest that *pvl*, *bbp*, and *sei* genes may provide as potential precautionary and diagnostic biomarkers for long-duration *S. aureus* OM, and therapeutic strategies targeting theses genes may help to shorten the OM infection duration.

Besides the crucial role of PVL in *S. aureus* OM mentioned above, we found some characteristics of PVL-positive OM cases and their isolates. Consistent with the result of a previous case control study enrolling *S. aureus* isolates from various infections (Boan et al., 2015), OM patients infected with PVL-positive *S. aureus* also tended to be younger (*p* = 0.012) and males (*p* = 0.01). But intriguingly, these characteristics are OM-specific in the current study (**Figures 5A–C**). The PVL-positive *S. aureus* strains were more likely to be *agr* group III/IV for OM patients, but not for non-OM patients (**Figure 5D**), this result suggests that OM infection may be more susceptible to *agr* group III/IV *S. aureus* carrying the *pvl* gene. In addition, we found that PVL-positive *S. aureus-*related OM patients showed more serious inflammatory responses reflected by significantly higher levels of CRP, WBC, and ANC values, and these significant differences were not found in non-OM patients infected with PVL-positive *S. aureus* (**Figures 4A,C,D**). Therefore, clinicians should be more vigilant against PVL-related OM when high values of these data were acquired from *S. aureus* OM patients. However, as the reason of functional redundancy, the lab experiments may not completely mean the actual function in the patient’s body, so further studies are still needed to verify the role of PVL in patients with *S. aureus* OM (Lebughe et al., 2017).

The present study has several limitations. First, the investigation was done in a single hospital. However, the data were representative because our hospital is one of the biggest hospitals in western China, taking patients from provinces mainly including Chongqing, Sichuan, Guizhou, and Yunnan. A multicentre study should be done in the future if possible. Second, not every patient’s laboratory data in the study could be obtained, but at least the data from 49/60 patients (for ESR data) in the OM group and 61/79 patients (for CRP data) in the non-OM group were collected, so it is able to reflect the tendency.

## Conclusion

Our study reveals that STs belonged to certain cluster are specific to *S. aureus* OM infection. While antimicrobial resistance is not responsible for prevalence or duration of *S. aureus* OM, some virulence factors play important roles in *S. aureus* OM. The *pvl*, *bbp*, and *ebps* genes may represent as risk factors for prevalence of *S. aureus* OM. Presence of *pvl*, *bbp*, and *sei* genes may prolong the duration of *S. aureus* OM. We also observed that PVL-related *S. aureus* OM showed more serious inflammatory responses. Our study suggests that PVL may play as a potential indicator for prevalence, duration, and severity of *S. aureus* OM. Special attention should be paid on PVL-related *S. aureus* OM in clinical diagnosis and treatment.

## Author Contributions

XH and ZX conceived and designed this study. BJ and YW carried out the experiments, collected, and analyzed the data. ZF, LX, LT, and SZ helped with the data collection and interpretation. YG, CZ, and XL helped with BioNumerics analysis and construction of phylogenetic tree. SL, XR, and YP drafted the manuscript. All authors have read and approved the final manuscript.

## Conflict of Interest Statement

The authors declare that the research was conducted in the absence of any commercial or financial relationships that could be construed as a potential conflict of interest.

## Footnotes

1 http://pubmlst.org/saureus/

2 http://spaserver.ridom.de
