# Supplementary material for: Panton-Valentine Leucocidin (PVL) as a Potential Indicator for Prevalence, Duration, and Severity of Staphylococcus aureus Osteomyelitis
Source: Front Microbiol. 2017 Nov 28;8:2355. doi: 10.3389/fmicb.2017.02355 (PMC5712352; doi:10.3389/fmicb.2017.02355)
Supplement: Supplementary file 1 [file Table_1.PDF]

Table S1. Primers used for virulence factors PCR

Gene name	Primer name	Nucleotide sequence(5’-3’)	Size(bp)
icaA	*icaA*-F	TGGCTGTATTAAGCGAAGTC	669
	icaA-R	CCTCTGTCTGGGCTTGACC	
bbp	*bbp*-F	AACTACATCTAGTACTCAACAACAG	573
	bbp-R	ATGTGCTTGAATAACACCATCATCT	
cna	*cna*-F	GTCAAGCAGTTATTAACACCAGAC	423
	cna-R	AATCAGTAATTGCACTTTGTCCACTG	
eno	*eno*-F	ACGTGCAGCAGCTGACT	302
	eno-R	CAACAGCATYCTTCAGTACCTTC	
ebps	*ebps*-F	CATCCAGAACCAATCGAAGAC	186
	ebps-R	CTTAACAGTTACATCATCATGTTTATCTTTG	
fnbA	*fnbA*-F	GTGAAGTTTTAGAAGGTGGAAAGATTAG	643
	fnbA-R	GCTCTTGTAAGACCATTTTTCTTCAC	
fnbB	*fnbB*-F	GTAACAGCTAATGGTCGAATTGATACT	524
	fnbB-R	CAAGTTCGATAGGAGTACTATGTTC	
fib	*fib*-F	CTACAACTACAATTGCCGTCAACAG	404
	fib-R	GCTCTTGTAAGACCATTTTCTTCAC	
clfA	*clfA*-F	ATTGGCGTGGCTTCAGTGCT	292
	clfA-R	CGTTTCTTCCGTAGTTGCATTTG	
clfB	*clfB*-F	ACATCAGTAATAGTAGGGGGCAAC	205
	clfB-R	TTCGCACTGTTTGTGTTTGCAC	

icaD	*icaD*-F	ATGGTCAAGCCCAGACAGAG	121
	icaD-R	CGAGTAGAACAACTAAACAATATATCC	
sen	*sen*-F	ATGAGATTGTTCTACATAGCTGCAAT	680
	sen-R	AACTCTGCTCCCACTGAAC	
sea	*sea*-F	GAAAAAAGTCTGAATTGCAGGGAACA	560
	sea-R	CAAATAAATCGTAATTAACCGAAGGTTC	
sed	*sed*-F	GAATTAAGTAGTACCGCGCTAAATAATATG	492
	sed-R	GCTGTATTTTTCCTCCGAGAGT	
seb	*seb*-F	ATTCTATTAAGGACACTAAGTTAGGGA	404
	seb-R	ATCCCGTTTCATAAGGCGAGT	
sec	*sec*-F	GTAAAGTTACAGGTGGCAAAACTTG	297
	sec-R	CATATCATACCAAAAAGTATTGCCGT	
seo	*seo*-F	AGTTTGTGTAAGAAGTCAAGTGTAGA	180
	seo-R	ATCTTTAAATTCAGCAGATATTCCATCTAAC	
seg	*seg*-F	AATTATGTGAATGCTCAACCCGATC	642
	seg-R	AAACTTATATGGAACAAAAGGTACTAGTTC	
sei	*sei*-F	CTCAAGGTGATATTGGTGTAGG	576
	sei-R	AAAAAACTTACAGGCAGTCCATCTC	
see	*see*-F	CAAAGAAATGCTTTAAGCAATCTTAGGC	482
	see-R	CACCTTACCGCCAAAGCTG	
seh	*seh*-F	CAATCACATCATATGCGAAAGCAG	376
	seh-R	CATCTACCCAAACATTAGCACC	

sej	*sej*-F	TAACCTCAGACATATATACTTCTTTAACG	300
	sej-R	AGTATCATAAAGTTGATTGTTTTCATGCAG	
tst	*tst*-F	TTCACTATTTGTAAAAGTGTCAGACCCACT	180
	tst-R	TACTAATGAATTTTTTTATCGTAAGCCCTT	
lukM	*lukM*-F	TGGATGTTACCTATGCAACCTAC	780
	lukM-R	GTTCGTTTCCATATAATGAATCACTAC	
etb	*etb*-F	CAGATAAAGAGCTTTATACACACATTAC	612
	etb-R	AGTGAACTTATCTTTCTATTGAAAAACACTC	
psmα	*psmα*-F	TTATTTTGCGAAAATGTCGATAATT	409
	psmα-R	ATGGGTATCATCGCTGGCATC	
sem	*sem*-F	TCATATCGCAACCGCTGATGT	350
	sem-R	GATACTTTGTCAGTAGATATAGTT	
lukED	*lukED*-F	TGAAAAAGGTTCAAAGTTGATACGAG	269
	lukED-R	TGTATTCGATAGCAAAAGCAGTGCA	
eta	*eta*-F	ACTGTAGGAGCTAGTGCATTTGT	190
	eta-R	TGGATACTTTTGTCTATCTTTTTCATCAAC	
edin	*edin*-F	GAAGTATCTAATACTTCTTTAGCAGC	625
	edin-R	TCATTTGACAATTCTACACTTCCAAC	
hlg	*hlg*-F	GTCAYAGAGTCCATAATGCATTTAA	535
	hlg-R	CACCAAATGTATAGCCTAAAGTG	
hlgv	*hlgv*-F	GACATAGAGTCCATAATGCATTYGT	390
	hlgv-R	ATAGTCATTAGGATTAGGTTTCACAAAG	

hlb	*hlb*-F	GTGCACTTACTGACAATAGTGC	309
	hlb-R	GTTGATGAGTAGCTACCTTCAGT	
hla	*hla*-F	CTGATTACTATCCAAGAAATTCGATTG	209
	hla-R	CTTTCCAGCCTACTTTTTTATCAGT	
hld	*hld*-F	AAGAATTTTTATCTTAATTAAGGAAGGAGTG	111
	hld-R	TTAGTGAATTTGTTCACTGTGTCGA	
pvl	*pvl*-F	A TCATTAGGTAAAATGTCTGGACATGATCCA	433
	pvl-R	GCATCAASTGTATTGGATAGCAAAAGC	
